# Plasma homocysteine concentrations and risk of intracerebral hemorrhage: a systematic review and meta-analysis

**DOI:** 10.1038/s41598-018-21019-3

**Published:** 2018-02-07

**Authors:** Zhike Zhou, Yifan Liang, Huiling Qu, Mei Zhao, Feng Guo, Chuansheng Zhao, Weiyu Teng

**Affiliations:** 1 Department of Geriatrics, The First Affiliated Hospital, China Medical University, Shenyang, 110001 Liaoning PR China; 2 Department of Neurology, The First Affiliated Hospital, China Medical University, Shenyang, 110001 Liaoning PR China; 3 3Department of Neurology, The People’s Hospital of Liaoning Province, Shenyang, 110016 Liaoning PR China; 4 Department of Cardiology, The Shengjing Affiliated Hospital, China Medical University, Shenyang, 110004 Liaoning PR China; 5 Department of Neurology, Fuxin Central Hospital, fuxin, 123000 Liaoning PR China

## Abstract

Intracerebral hemorrhage (ICH) has the highest mortality rate in all strokes. However, controversy still exists concerning the association between plasma homocysteine (Hcy) and ICH. A systematic review and meta-analysis was conducted using Pubmed, Embase, and Web of Science up to April 18, 2017. Standard mean difference (SMD) for mean differences of plasma Hcy levels with 95% confidence intervals (CI) was calculated. Seven studies including 667 ICH patients and 1821 ischemic stroke patients were identified for meta-analysis. Our results showed that Hcy levels in ICH patients were significantly higher than those in healthy controls (SMD = 0.59, 95% CI = 0.51–0.68, P < 0.001); no statistic differences were found in the comparisons of Hcy levels between ICH and ischemic stroke (SMD = −0.03, 95% CI = −0.13–0.06, P > 0.05); further subgroup analysis of ethnicity (Asians: SMD = 0.57, 95% CI = 0.48–0.66, P < 0.001; Caucasians: SMD = 0.77, 95% CI = 0.51–1.02, P < 0.001) and sample size (small samples: SMD = 0.55, 95% CI = 0.30–0.80, P < 0.001; large samples size: SMD = 0.60, 95% CI = 0.51–0.69, P < 0.001) in relation to Hcy levels between ICH and healthy controls did not change these results. In conclusion, Hcy level may be an aggravating factor in atherosclerosis, which is positively associated with high risk of ICH. Race-specific differences between Asians and Caucasians have no impact on the risk of ICH.

## Introduction

Intracerebral hemorrhage (ICH), which accounts for 10–15% of all strokes, is the second most detrimental subtype of stroke^1,2^. ICH is more severe and disabling than ischemic stroke; it has the highest mortality rate of all the strokes^3,4^. Though our understanding of ICH has advanced in recent decades, so far this knowledge has not been translated into significant improvement in efficient therapies and prognosis^5–7^. Therefore, emphasis has been placed on preventive medicine aiming at reducing traditional risk factors of ICH, mainly hypertension, hyperlipidemia, diabetes mellitus and smoking^8–11^. A high level of homocysteine (Hcy) has been regarded as a qualifiable risk factor for ischemic stroke, and Hcy-lowering therapy is widespread used for those patients^12,13^. According to guidelines for healthcare professionals from the American Heart Association/American Stroke Association, though no randomized, placebo-controlled study has shown that Hcy-lowering therapy reduces ischemic stroke recurrence, it is recommended that supplements of folic acid, vitamin B6 and vitamin B12 reduce levels of homocysteine and may be considered for patients with ischemic stroke and hyperhomocysteinemia^14^. However, the relationship between Hcy and ICH remains controversial. Preview study showed no obvious association between Hcy level and risk of hemorrhagic stroke^15^. Recently, some studies revealed that patients with ICH exhibited higher concentrations of Hcy than that of healthy controls, which implied an underlying mechanism linking plasma Hcy levels with ICH^16–18^.

Homocysteine, an intermediate sulfhydryl-containing amino acid, is synthesized by the liver in a reaction involving the removal of the methyl group from methionine^19,20^. High level of Hcy is mainly found in patients with a nutritional deficiency of B vitamins as well as being present in genetic diseases where there is an insufficient level of cystathionine beta-synthase enzyme (CβS) or a mutation inn the enzyme 5,10-methylenetetrahydrofolate reductase (MTHFR). Other factors can elevate the level of Hcy i.e. a diet with high methionine content, smoking and a sedentary lifestyle^21–24^. According to epidemiological evidence, Hcy is considered to be a potential predictor of pathophysiology of neuronal system disorders^25,26^. High level of Hcy induces a neuro-inflammatory process due to its abilities to react with cytokines in the brain, leading to increased permeability of blood-brain barrier (BBB) and impaired integrity of cerebral vessels^27,28^. There is convincing evidence that elevated plasma Hcy level is associated with higher mortality rates from ischemic stroke, cardiovascular diseases and peripheral artery disease^29–32^. However, it is still controversial whether elevated plasma Hcy level is a risk factor for ICH. Therefore, we performed a systematic overview of retrospective studies to ascertain the association between Hcy and the risk of ICH.

## Materials and Methods

### Inclusion and Exclusion Criteria

To identify eligible studies investigating the association between plasma Hcy levels and ICH, published studies meeting the following criteria were considered eligible for inclusion: (1) case-control studies written in English; (2) studies which assessed the correlation between Hcy and patients with ICH; (3) the cases were confirmed ICH patients and the controls were healthy people; (4) providing information of plasma Hcy levels in the control group and case group at the onset of ICH. The exclusion criteria were as follows: (1) conflicts with the inclusion criteria; (2) publications with duplication or studies with overlapping data from the same study; (3) abstracts, case report, proceedings, letters, reviews, or meta-analysis.

### Literature Search

Databases of Pubmed, Embase, and Web of Science were comprehensively searched for articles up to April 18, 2017. The terms searched were as follows: (“plasma homocysteine” OR homocysteine OR Hcy OR “plasma Hcy”) AND (“intracerebral hemorrhage” OR “cerebral hemorrhage” OR “brain hemorrhage” OR “hemorrhagic stroke”). We also screened the reference lists of relevant reviews and included relevant articles in these lists.

### Data Extraction and Quality Assessment

Two reviewers independently extracted the data and information from the finally selected trials according to a standard protocol, and then discussed or consulted the original report to reach a consensus when discrepancies occurred. In the case of eligible studies, the following information was collected: first author, publication year, country, detection method for plasma Hcy, proportion of men, number of participants as well as plasma Hcy levels in the groups of ICH, control and ischemic stroke. Guidelines of preferred reporting items for systematic reviews and meta-analyses (PRISMA) were followed by the study. Quality assessment was conducted according to the Newcastle-Ottawa Scale (NOS) criteria for retrospective studies^33^. A score of up to 9 points was assigned to each study: 4 points for selection, 2 points for comparability, and 3 points for assessment of outcomes. Studies with 6 or more points were considered as high quality, while those with less than 6 were considered to be of suboptimal quality.

### Statistical Analysis

Statistical analysis in this meta-analysis was performed with the software Review Manager 5.2 and STATA version 14.0. Standard mean difference (SMD) with 95% confidence intervals (CI) was calculated by random effects model or fixed effects model to measure the differences in plasma Hcy levels between the case group and control group, the significance of which was assessed by utilizing the Z test. I^2^ test (25, 50, and 75% represented low, moderate, and high degrees of heterogeneity) was applied to assess the heterogeneity among studies^34–36^. Random effects models were applied for the evidence of significant heterogeneity (I^2^ test exhibited > 50%); otherwise, the fixed effects model was utilized^34,35,37^. Hcy levels from plasma and serum were used equally. Subgroup analyses by ethnicity and sample size of participants (≥50 or less) were also conducted. Sensitivity analysis was carried out by sequentially omitting one study to examine its influence on the pooled risk estimates. A publication bias was detected by visual inspection of a funnel plot i.e. an asymmetric funnel plot indicated a possible risk of a publication bias.

## Results

### Study selection and characteristics

**Figure 1:**
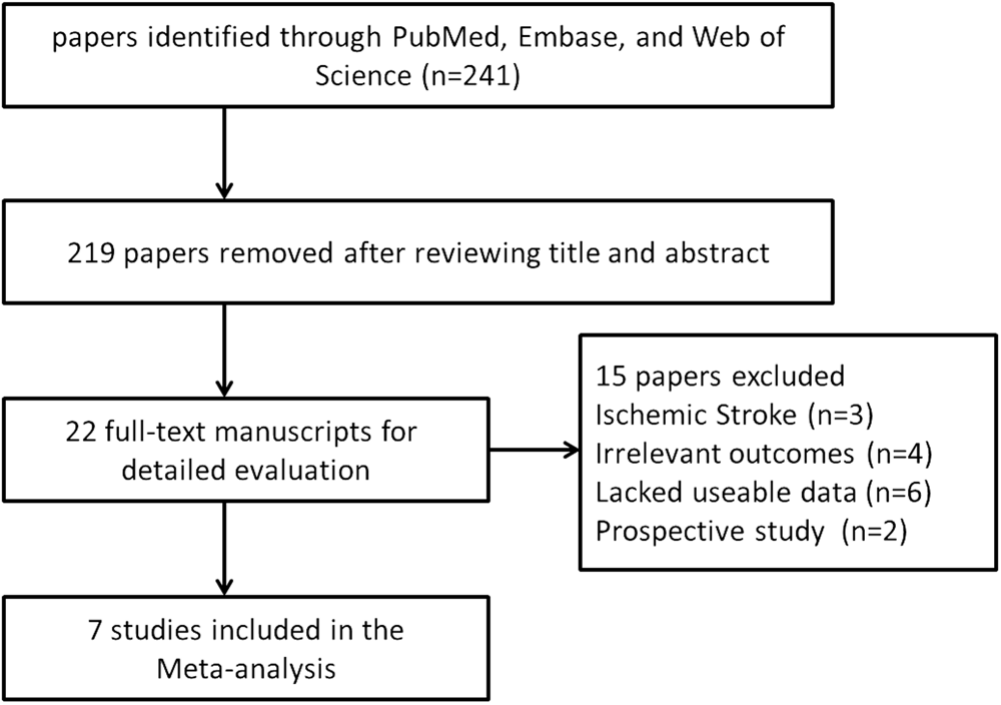
Flow chart of study selection in the meta-analysis.

A total of 241 abstracts were retrieved initially. After exclusion of 219 irrelevant or duplicated reports, 22 potentially eligible studies were assessed by reviewing the full text^15–18,38–55^. In all, 15 studies were further excluded, including 4 studies with irrelevant outcomes, 3 studies containing ischemic stroke patients, 6 studies in which there was a lack of usable data and 2 prospective studies. Thus, 7 studies were finally included into the current meta-analysis^15,50–55^ (Fig. 1). According to the NOS criteria (Table 1), these seven studies were categorized as high quality. The main characteristics of the studies included in the meta-analysis are detailed in Table 2. Those 7 studies involved a total of 667 patients with ICH, 1821 patients with ischemic stroke and 2500 healthy controls^15,50–55^. In six of the seven trials, the study subjects were Asians, the seventh trial was performed in Caucasians. When examining the sample sizes, 2 studies with large samples (n ≥ 50) whereas the other five studies examined small samples (n < 50). Figure 1 shows the flowchart of the study selection process and the screening steps.

**Table 1:** The Newcastle-Ottawa Quality Assessment Scale for case-control studies.

Study	Selection	Comparability	Outcome	Summary
Bokhari FA (2012)	4	2	2	8
Hiroyasu I (2004)	3	2	2	7
Fang X (2005)	4	2	2	8
Li ZH (2003)	4	2	2	8
Araki A (1989)	4	2	2	8
Perini F (2005)	4	2	2	8
Peng H (2000)	4	2	2	8

**Table 2:** General characteristics of the included studies.

First author	Year	Country	Detecting methods	Hemorrhagic			Controls			Ischemic		
				No. of male patients	tHcy, μmol/L Mean ± SD	Age, Years	No. of male patients	tHcy, μmol/L Mean±SD	Age, Years	No. of male patients	tHcy, μmol/L Mean±SD	Age, Years
Bokhari FA	2012	Pakistan	ELISA	4/9	23.73 ± 13.61	NR	NR/33	20.73 ± 8.59	NR	10/22	20.45 ± 9.47	NR
Hiroyasu I	2004	Japan	HPLC	17/38	10.4 ± 2.86	64.7	51/114	9.2 ± 3.1	64.4	60/98	9.8 ± 3.99	65.9
Fang X(+/−)	2005	Mongolia	HPLC	NR/12	15.6 ± 4.6	NR	NR/6	12.9 ± 5.3	NR	NR/18	18.4 ± 8.9	NR
Fang X(+/+)	2005	Mongolia	HPLC	NR/5	20 ± 6.4	NR	NR/2	15.7 ± 6.3	NR	NR/11	21.3 ± 1.8	NR
Li ZH	2003	China	HPLC	318/503	14.6 ± 1.3	58.2 ± 9.6	1052/1832	12.8 ± 3.47	59.6 ± 8.8	513/807	14.7 ± 8.6	61.3 ± 9.7
Araki A	1989	Japan	HPLC	13/20	9.6 ± 3.9	65.4 ± 9.7	30/45	7.3 ± 2.9	62.9 ± 10.8	30/45	13.1 ± 5.6	63.3 ± 10.9
Perini F	2005	Italy	HPLC	39/70	18.18 ± 14.08	69.5 ± 13.8	201/421	11.97 ± 6.61	56.6 ± 14.8	399/775	18.94 ± 13.65	71.6 ± 12.1
Fang X(−/−)	2005	Mongolia	HPLC	NR/3	20.5 ± 8.6	NR	NR/16	15.4 ± 4.2	NR	NR/13	18.9 ± 8.0	NR
Peng H	2000	China	HPLC	NR/7	18.77 ± 5.58	NR	26/31	11.46 ± 5.96	51.8	NR/32	18.72 ± 10.55	NR

M male, n number, tHcy total homocysteine, NR not reported, ELISA enzyme-linked immunosorbent assay, HPLC high-performance liquid chromatography, +/+ homozygous methylenetetrahydrofolate reductase gene mutation, +/− heterozygous methylenetetrahydrofolate reductase gene mutation, −/− wild-type.

### Meta-analysis

**Figure 2:**
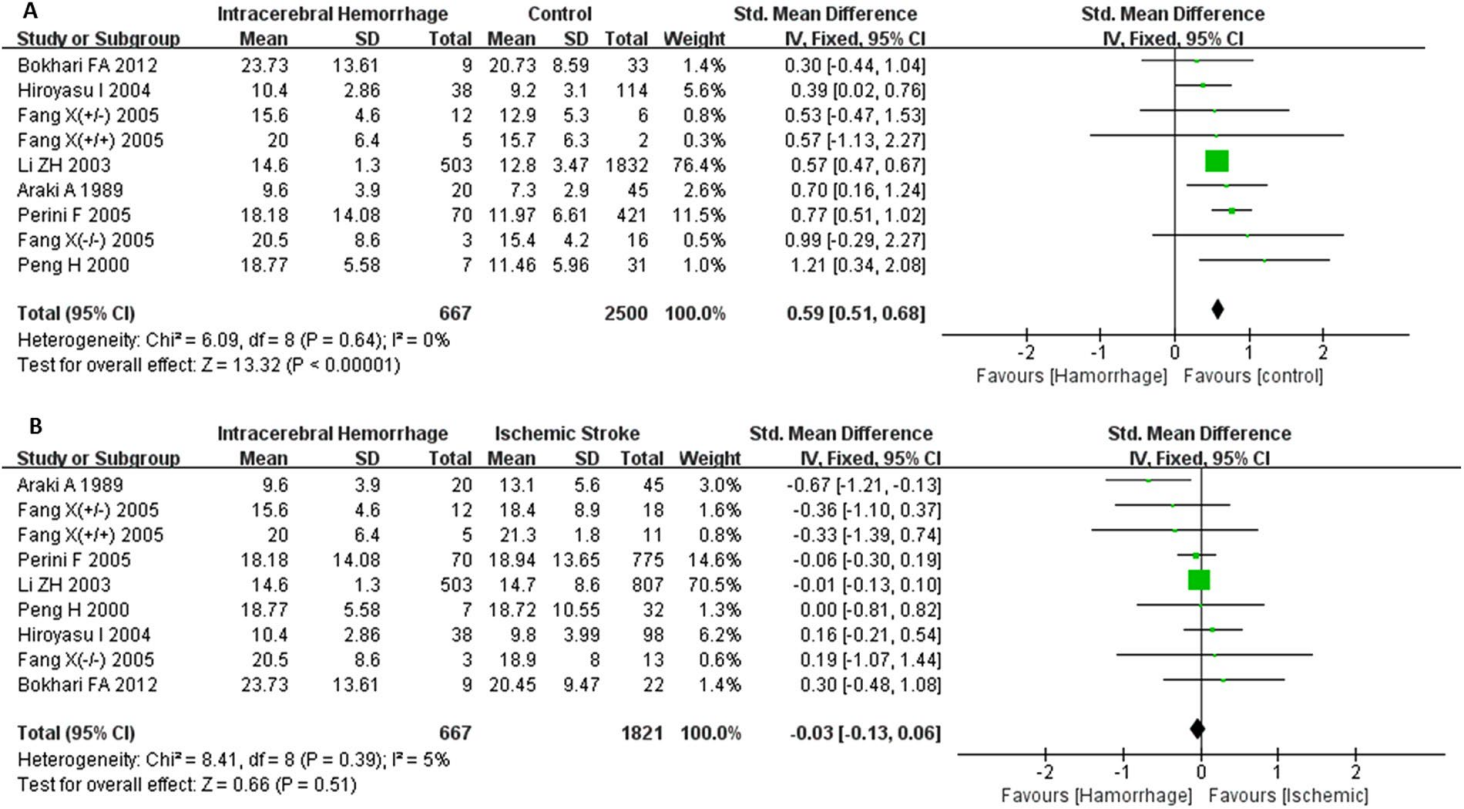
Forest plots for the comparisons of plasma Hcy levels between intracerebral hemorrhage (ICH) patients and healthy controls (**A**); Forest plots for the comparisons of plasma Hcy levels between intracerebral hemorrhage (ICH) patients and ischemic stroke patients (**B**). CI: confidence interval.

**Figure 3:**
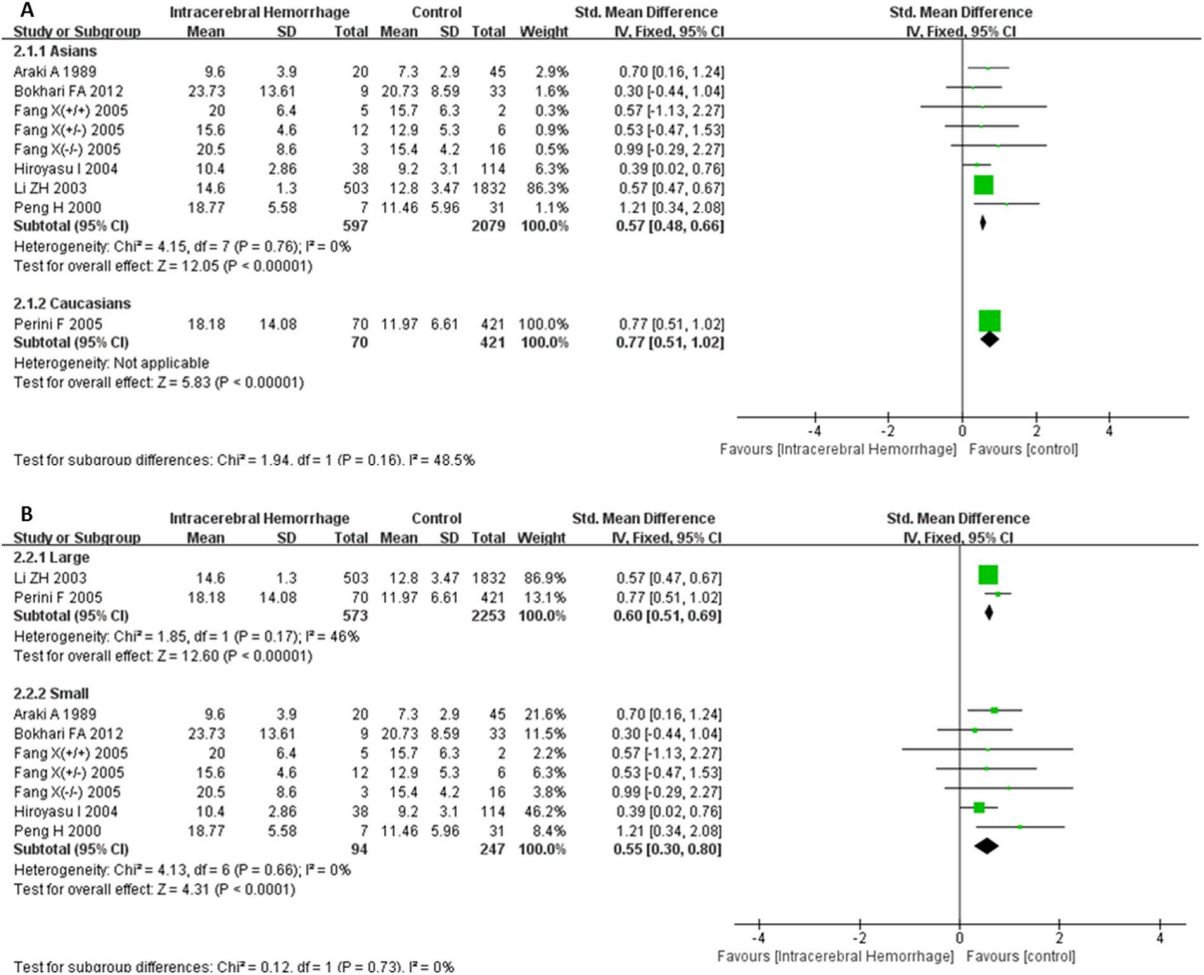
Forest plots of the subgroup analyses on ethnicity (**A**) and sample size (**B**) in relation to plasma Hcy levels between intracerebral hemorrhage (ICH) patients and healthy controls. CI: confidence interval.

There was no heterogeneity among those 7 studies reporting differences of plasma Hcy levels between groups of ICH and healthy controls (I^2^ = 0%), as well as groups of ICH and ischemic stroke (I^2^ = 5%); thus, the fixed-effects model was used to pool data. Patients with ICH had a higher serum Hcy level compared with control group (SMD = 0.59, 95% CI = 0.51–0.68, P < 0.001) (Fig. 2A). There were no significant differences in the comparisons of plasma Hcy levels between ICH group and ischemic stroke group (SMD = −0.03, 95% CI = −0.13–0.06, P > 0.05) (Fig. 2B). The results of subgroup analysis based on ethnicity indicated that the plasma Hcy levels of ICH patients were significantly higher than those of healthy controls in both Asians and Caucasians (Asians: SMD = 0.57, 95% CI = 0.48–0.66, P < 0.001; Caucasians: SMD = 0.77, 95% CI = 0.51–1.02, P < 0.001), and there were no statistical differences between those two groups (P > 0.05) (Fig. 3A). A further subgroup analysis on sample size revealed that ICH patients displayed higher plasma Hcy levels than healthy controls in both small samples (n < 50) and large samples (n ≥ 50) (small samples: SMD = 0.55, 95% CI = 0.30–0.80, P < 0.001; large samples size: SMD = 0.60, 95% CI = 0.51–0.69, P < 0.001), and there were no statistical differences between those two groups (P > 0.05) (Fig. 3B).

### Sensitivity Analysis and Publication bias

**Figure 4:**
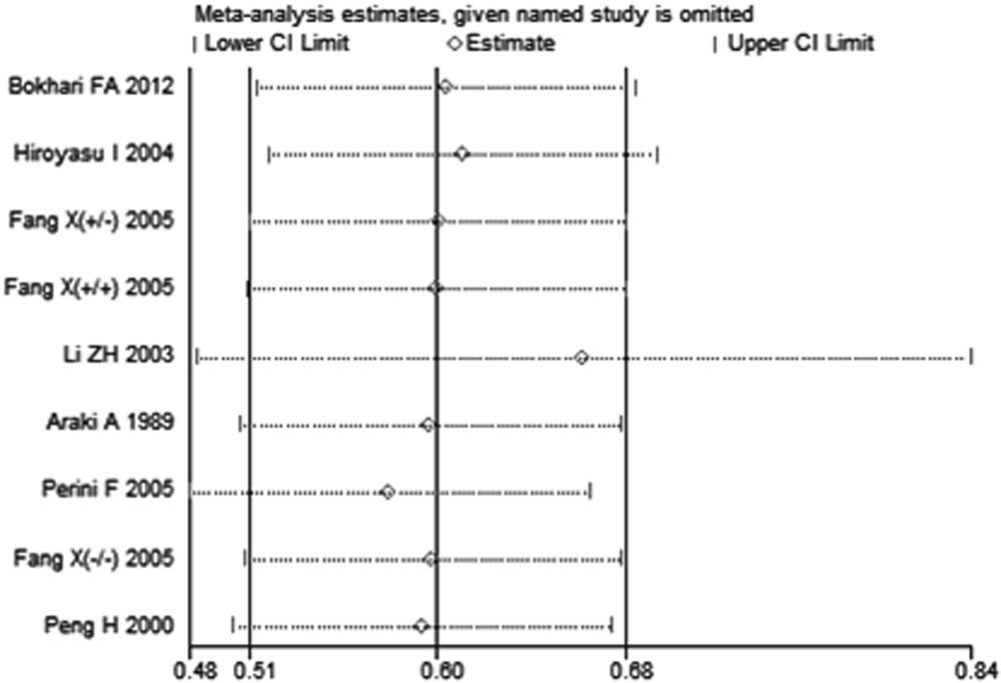
Forest plot in the sensitivity analysis of the present meta-analysis (individual names for each study have been omitted). CI: confidence interval.

**Figure 5:**
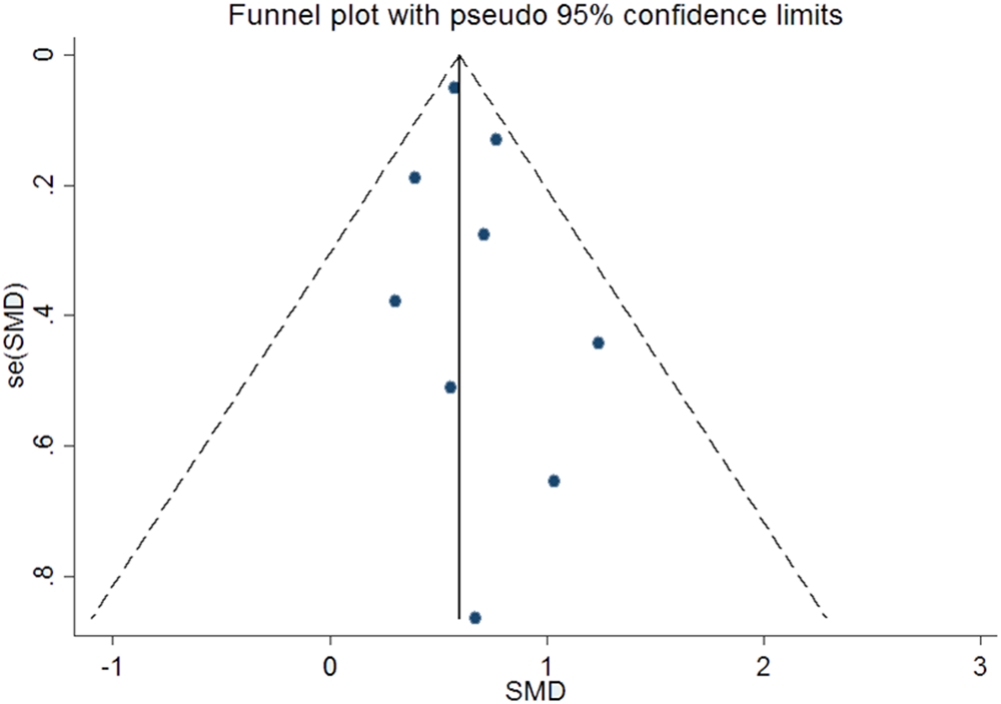
Funnel plot to detect risk of publication bias in the meta-analysis. SMD: standard mean difference.

Sensitivity analysis performed by omitting one individual study sequentially showed that no single study had any obvious influence on the pooled mean difference (Fig. 4). Since the funnel plot for overall analysis was symmetrical (Fig. 5), there was no obvious risk of publication bias in the meta-analysis.

## Discussion

As far as we are aware, there has been no relevant systematic review of case-control studies carried out to explore whether plasma Hcy are associated with the risk of ICH. Seven eligible studies with a total of 667 ICH patients were included into our meta-analysis^15,50–55^. In comparison with a single study, the meta-analysis makes it possible to have a more accurate estimate and evaluation of the impact of Hcy levels on ICH. The findings emerging from comprehensive meta-analysis confirmed that patients with ICH exhibited prominently higher plasma Hcy levels than healthy controls, and this association could not be affected by study quality, participant characteristics and publication bias.

The precise mechanism of Hcy on the susceptibility of ICH remains unresolved. However, our meta-analysis supported that the pathogenesis of ICH likely involves some factor elevating Hcy levels. Experimental studies have verified multiple biological responses triggered by Hcy during the process of atherosclerosis, for example, Hcy inhibits glutathione peroxidase to stimulate the proliferation of smooth muscle cells, by which endothelial nitric oxide synthase (eNOS) can be suppressed via the asymmetric dimethyl-L-arginine (ADMA) pathway, leading to endothelial dysfunction and vascular spasms^26,56–58^. In addition, Hcy enhances the release of arachidonic acid from platelets to generate reactive oxygen species (ROS), leading to the deposition of calcium and lipid in the endothelial wall; these changes degrade arterial elasticity and accelerate the process of atherosclerosis^21,59–61^. In the present results, the comparisons of plasma Hcy levels between ICH and ischemic stroke showed no significant difference. This implied that the plasma Hcy level was likely identified as an aggravating factor in atherosclerosis, owing to which the pathophysiology of endothelial degeneration and necrosis of vessel walls could elevate the risk of ischemic stroke and cerebral hemorrhage^62^.

However, we are unable to rule out the possibility that the high levels of Hcy may be attributable to the acute stress reaction evoked by stroke, as all blood samples of plasma Hcy were obtained at the onset of ICH rather than before its occurrence. There is one prospective study in which participants were invited to donate a fasting blood sample for future research; this showed that plasma Hcy levels were significantly higher in patients who would develop ICH compared to their healthy controls^49^. Another study reported that Hcy levels initially decreased within 24 hours and then gradually raised within 48 hours in patients with hemorrhagic stroke, whereas differences in mean Hcy levels at 0, 24 and 48 hours post stroke were insignificant compared with mean Hcy levels in controls^54^. Accordingly, it is speculated that Hcy levels have been already elevated before the onset of ICH and that their levels could not be affected by acute stress reaction after a stroke. Further studies will be needed to assess the influence of the acute stress reaction during follow-up on the association between Hcy and ICH.

Consistent with the overall results, subgroup analysis on either ethnicity or sample size confirmed that plasma Hcy levels of ICH patients were significantly higher than those of healthy controls. Preview study revealed that Africans had apparently higher risk of ICH compared with Caucasians, suggesting that race played an important role in the ICH risk pattern^63^. Interestingly, in the present study, the subgroup analysis based on ethnicity in relation to plasma Hcy levels between Asians and Caucasians had no statistic differences, which implied that race-specific differences exerted no effect on the results of our meta-analysis. The reason for this inconsistency might be that race-specific differences between Africans and Caucasians had a profound impact on the risk of ICH rather than race-specific differences between Asians and Caucasians in our study. Although our meta-analysis revealed no statistical heterogeneity, there can also be clinical heterogeneity or methodological heterogeneity between studies. Some possible sources of clinical heterogeneity such as inclusion and exclusion criteria, intervention measures, defining indicators may be the differences. Other sources of methodology heterogeneity such as experimental design, bias risk and outcome integrity may also affect the results.

### Limitations

Several potential limitations of this meta-analysis must be acknowledged. All included studies followed a retrospective design, which may be more subjected to bias and artifact than prospective studies. The different detection methods employed for plasma Hcy levels may have sensitivity and reliability issues. We were unable to analyze the effect of the acute stress reaction of ICH on plasma Hcy levels since there were insufficient data about Hcy levels before the onset of ICH. Although the result of the funnel plot did not suggest any publication bias, there is the possibility of journals publishing only positive findings as only published data were utilized in the meta-analysis. Additionally, the relatively limited number of studies and the fact that most of patients were Asians, may also influence the sensitivity of statistical analysis. Further randomized controlled trials and larger numbers of participants will be necessary to confirm these findings.

## Conclusions

Our results of meta-analysis provide support for the hypothesis that elevated level of Hcy in blood is positively associated with the incidence of ICH. Hcy level may be an aggravating factor in atherosclerosis which contributes to ICH. There is no impact of race-specific differences between Asians and Caucasians on the risk of ICH. More clinical trials with more comprehensive data, using larger sample sizes and better design, will be needed to validate our results.
